# Supplementary material for: A simple method for detecting chaos in nature
Source: Commun Biol. 2020 Jan 3;3:11. doi: 10.1038/s42003-019-0715-9 (PMC6941982; doi:10.1038/s42003-019-0715-9)
Supplement: Supplementary file 1 — Supplementary Information [file 42003_2019_715_MOESM1_ESM.pdf]

SUPPLEMENTARY FIGURES

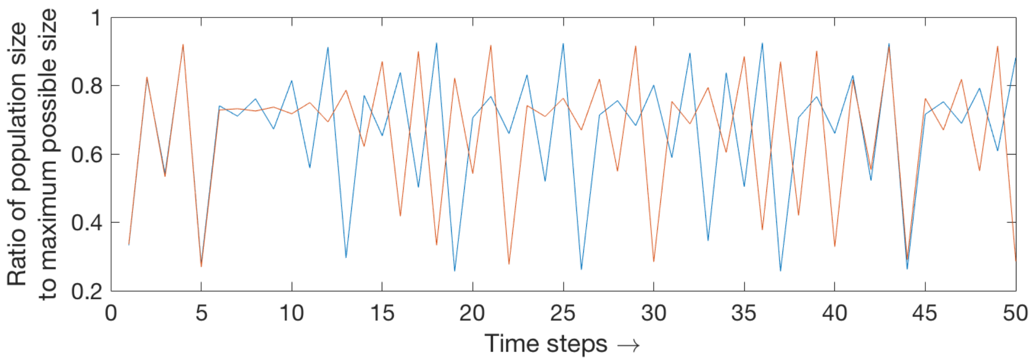

Supplementary Figure 1: A graphical depiction of chaos. Here, we show two simulations of the chaotic logistic map, which is a simple model of population dynamics (see Methods). The starting values of the two simulations are only very slightly different (they differ by a value of only 0.001), and yet by the fifth time-step, the two simulations show a clear divergence in their trajectories. This extreme sensitivity to small perturbations (within bounded, predominantly deterministic systems) is what it means to be chaotic.

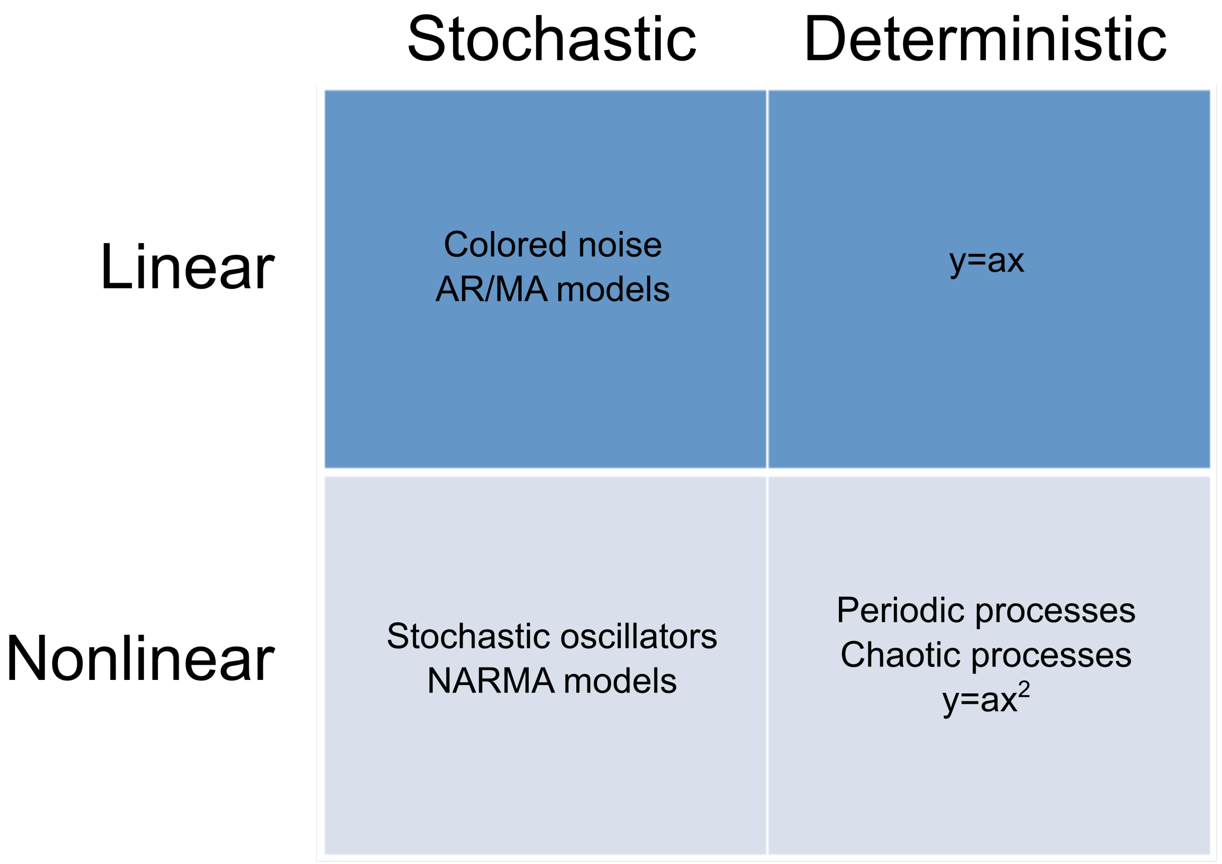

Supplementary Figure 2: Dynamical systems can be broadly categorized as linear or nonlinear, and as deterministic or stochastic. Both chaotic and periodic processes are nonlinear and deterministic, but chaotic processes have a positive largest Lyapunov exponent (meaning that initially similar system states diverge exponentially fast) and periodic processes have a negative largest Lyapunov exponent.

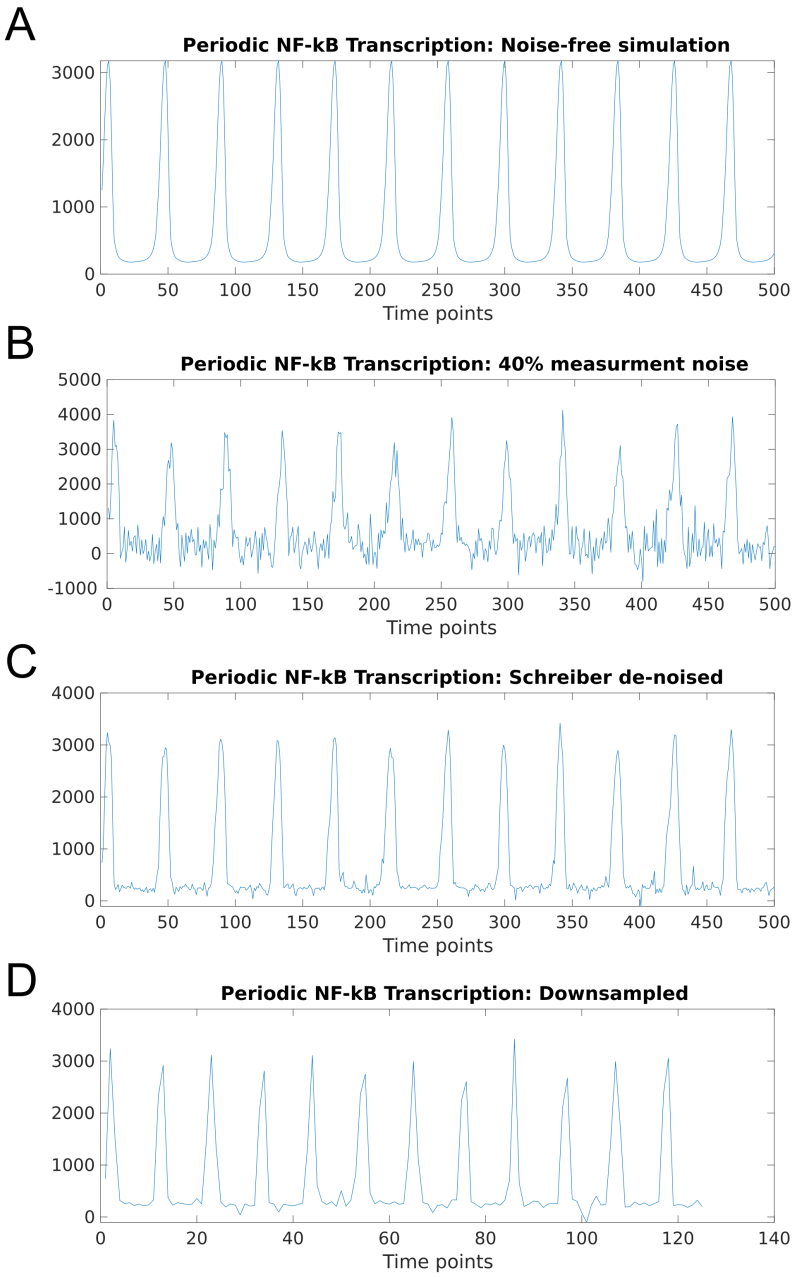

Supplementary Figure 3: Sample time-series to illustrate the steps of the Chaos Decision Tree Algorithm. **A** The first 500 time-points of a noise-free simulation of periodic NF-κB transcription (out of 10,000). **B** The same time-series as in **A**, but with added white noise, the amplitude of which is 40% the standard deviation of the original time-series. **C** The same time-series as in **B**, but after Schreiber de-noising. **D** The same time-series as in **C**, but after the de-noised signal has been iteratively downsampled until the difference between the global maximum and global minimum of the signal, divided by the mean absolute difference between consecutive time-points in the signal, is less than or equal to 10. The 0-1 test is applied after this final step.

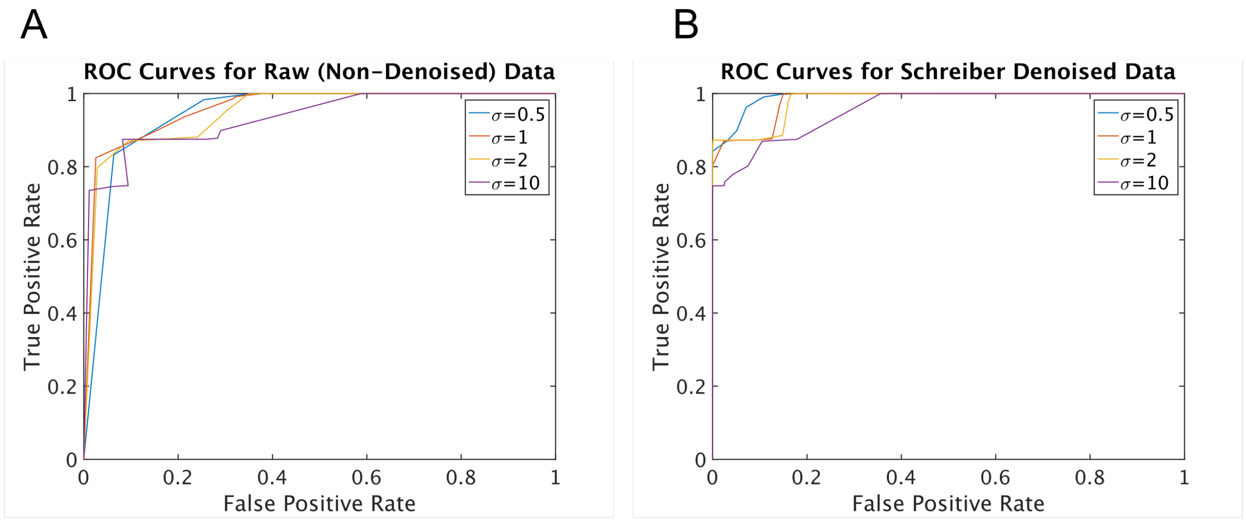

Supplementary Figure 4: Receiver Operating Characteristic Curves for different parameters of the 0-1 chaos test, for both raw data (A) and Schreiber de-noised data (B). Here, we included all deterministic datasets from Tables 1 and 2. Systems were classified as periodic or chaotic based on different cutoffs of the K-statistic; cutoffs were K=0.1, K=0.5, K=0.55, K=0.6, K=0.65, K=0.7, K=0.75, K=0.8, K=0.85, K=0.9, K=0.95, K=0.96, K=0.97, K=0.98, and K=0.99. We also varied the parameter σ in the modified 0-1 test, which controls the amplitude of a noise term that is used to suppress correlations arising from quasi-periodicity (and thus improves classification of strange non-chaotic systems as periodic) - see Eq. 3 in the Methods. Across all datasets and noise levels, the 0-1 test with $\sigma = 0.5$ applied to Schreiber denoised data provided the highest classification accuracy, as can be seen by the large area under the curve in B.

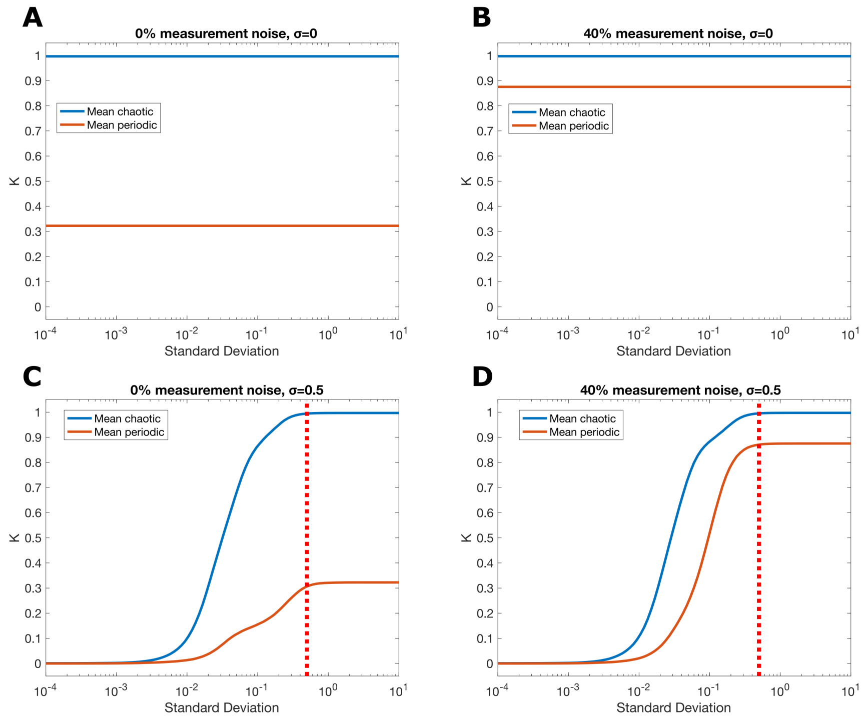

Supplementary Figure 5: We found that inclusion of a noise term σ in the modified 0-1 test for chaos (see Eq. 3 in Methods) can lead to inaccurate results for signals with very low amplitude/standard deviation. To illustrate this effect, we ran the modified 0-1 test on a single simulation, with either 0% or 40% measurement noise, of each deterministic process in Tables 1-2. To vary standard deviation, we multiplied each simulation by a constant, such that its standard deviation was fixed at a given value, out of 100 values logarithmically spaced between 10^{-4} and 10^{1} . We then calculated the K-statistic (i.e. the output of the modified 0-1 test) for both σ=0 (**A**-**B**) or σ=0.5 (**C**-**D**). Here, we plot the mean K-statistic across all chaotic systems (blue), as well as the mean K-statistic across all periodic systems (red), as a function of signal standard deviation. For σ =0, K was invariant to the standard deviation of the signal, for either 0% measurement noise (**A**) or 40% measurement noise (**B**). For σ =0.5, on the other hand, K fell to zero for both periodic or chaotic signals as their standard deviation approached zero, for both 0% measurement noise (**C**) and 40% measurement noise (**D**). This is likely because the inclusion of a non-zero noise term in Eq. 3 of the 0-1 test (see Methods) overwhelms the mean squared displacement of signals with very small standard deviations. Note that for σ =0.5, K asymptotes at around a standard deviation of 0.5 for both levels of measurement noise. As such, we modified the 0-1 test to normalize the standard deviation of a test signal to 0.5 (see Methods).

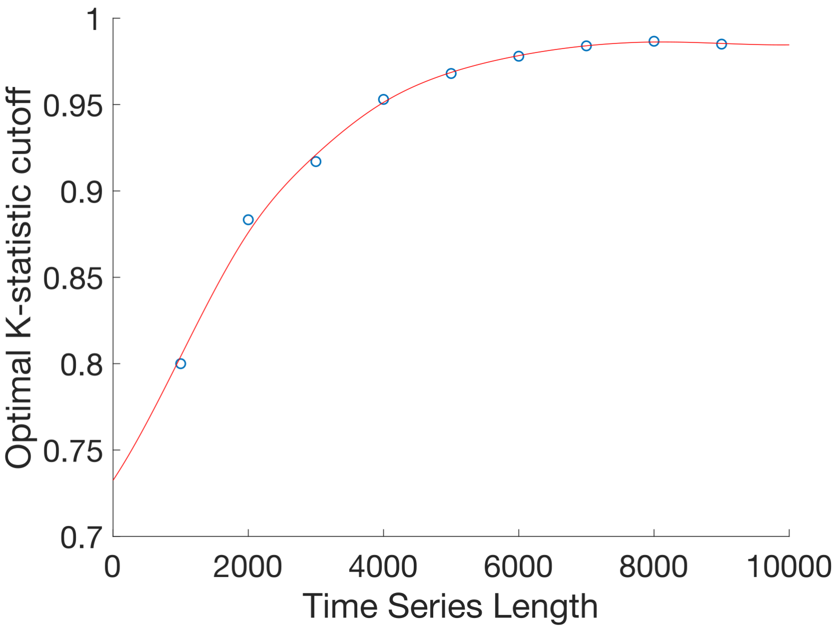

Supplementary Figure 6: Though all analyses in the main body of the paper were performed on time-series with 10,000 time-points, we also considered how to optimize the algorithm's performance for shorter time-series. In particular, it is known that the K-statistic of the 0-1 test approaches 1 for chaotic systems and approaches 0 for periodic systems *as* the length of a time-series is increased, but that it can yield intermediate - and therefore ambiguous - results for shorter time-series. As such, we ran the chaos-testing portion of our algorithm's pipeline (which consists of signal de-noising, then normalizing the standard deviation of the signal, then applying the 0-1 test, and then classifying the system as either periodic or chaotic based on some cutoff of the outputted K-statistic) on sub-samples of all non-oversampled deterministic datasets in Tables 1 and 2, including all levels of measurement noise. We did not include signals classified as oversampled in this analysis, as the downsampling step of our algorithm would shorten the length of the time-series, which would warp the relationship between the optimal K-statistic cutoff and time-series length. We ran the pipeline on samples ranging from 1,000 to 9,000 time-points in length, in intervals of 1,000. For each time-series length, we calculated the F1 score of different K-statistic cutoffs, ranging from K=0.005 to K=0.995, in steps of 0.005. Here, we plot the smoothed vector of optimal K-statistic cutoffs for each time-series length. As expected, given the fact that the K-statistic *approaches* 1 for chaotic signals as time-series length is increased, the optimal cutoff also increased as a function of time-series length, asymptoting near K=0.985 for longer time-series. Thus, if no cutoff is provided to the algorithm, it will automatically pick a cut-off based on the smoothing spline fit plotted here in red. If the predicted optimal cutoff is greater than 0.99, the algorithm picks a cutoff of 0.99 (since the K-statistic is upper-bounded by 1). With this automated cutoff selection, we have confirmed that the Chaos Decision Tree Algorithm performs at very high accuracy for time-series with 1,000 points (Supplementary Table 16), 5,000 points (Supplementary Table 17), and 10,000 points (Tables 1-3).

SUPPLEMENTARY TABLES

Supplementary Table 1: Our algorithm uses surrogate data methods to test for stochasticity (Fig. 1, Supplementary Tables 2-3), but other studies emphasize the importance of first testing for stationarity before using such methods[1,2] (due to the inherent stationarity of Fourier-based surrogates). A stationary signal is one whose unconditional joint probability distribution is time-invariant. We here assess the performance of a number of stationarity tests (see Patterson[3,4] for a thorough review of such tests and their relative strengths/weaknesses). Datasets analyzed include all stationary processes in Tables 1-2 (including bounded random walks, autoregressive processes with moving averages, and nonlinear stochastic processes, all of which are difficult edge cases for such tests[3,4]), all unit root processes in Table 2 (including random walks and trended random walks), and a cyclostationary autoregressive process. See Methods for details on all datasets. The stationarity tests we assessed are the augmented Dickey-Fuller (ADF) test[5], the Kwiatkowski–Phillips–Schmidt–Shin (KPSS) test[6], and the Leybourne-McCabe (LMC) test[7], and the non-parametric Lo and MacKinlay Variance Ratio[8,9] (LM-VR) and Breitung's Variance Ratio[10] (BVR) tests, all with and without first detrending the test signal. Shown: fraction of datasets classified as non-stationary, and F1 scores for stationary vs. unit root processes. Consistent with the analyses performed by Patterson[3,4], we found that Breitung's Variance Ratio (BVR) test significantly outperformed other tests, though the poor performance for the cyclostationary process underscores the fact that a unit root is only one form of non-stationarity.

	Stationary	Unit Root	Cyclostationary	F1
ADF test	506/10500	242/1000	0/500	0.28
KPSS test	2850/10500	1000/1000	0/500	0.41
LMC Test	2172/10500	903/1000	0/500	0.44
LM-VR test	215/10500	187/1000	0/500	0.27
BVR test	161/10500	961/1000	0/500	0.91
ADF test (detrended)	6/10500	76/1000	0/500	0.14
KPSS test (detrended)	2850/10500	1000/1000	0/500	0.41
LMC Test (detrended)	2172/10500	903/1000	0/500	0.44
LM-VR test (detrended)	215/10500	187/1000	0/500	0.27
BVR test (detrended)	161/10500	935/1000	0/500	0.89

Supplementary Table 2: F1 scores for surrogate-based tests of stochasticity. For each test dataset, 500 surrogates were generated using Lancaster and colleagues' Matlab toolbox[1]. For all surrogate algorithms, the data are first pre-processed such that the start and end points of the data and their first derivatives are matched as closely as possible. We used permutation entropy as our test statistic, so that a signal was classified as stochastic if its permutation entropy fell within the distribution of permutation entropies calculated from the 500 surrogate time-series. We tested the effect of Schreiber denoising ("Denoised") vs. no denoising ("Raw") on test accuracy. We further tested the effect of normality transformation using the Box-Cox method ("Transformed (Box-Cox)"), following the recommendation of Chan and Tong[11], or using a rank-based inverse normal transformation ("Transformed (INT)"), vs. no normality transformation ("Non-transformed"). Moreover, we tested the benefit of excluding signals classified as non-stationary by Breitung's Variance Ratio test ("Stationary Data") vs. including all signals ("All data"). We assessed five different surrogate algorithms, namely Amplitude Adjusted Fourier Transform[12] (AAFT) surrogates, Fourier Transform[12] (FT) surrogates, iterative Amplitude Adjusted Fourier Transform[12] (iAAFT) surrogates, Cycle Shuffled Surrogates[13] (CSS), and Cyclic Phase Perutation[14] (CPP) surrogates, as well as a combination of Amplitude Adjusted Fourier Transform and Cyclic Phase Permutation (AAFT+CPP) surrogates (see Methods). Finally, we tested eight different permutation order values for the calculation of permutation entropy, which was the test statistic used to discriminate between the original and surrogate signals. Stochastic datasets consisted of all linear and nonlinear stochastic processes in Tables 1-2, and deterministic datasets consisted of all deterministic processes in Tables 1-2, which included all datasets with added measurement noise. Performance was highest for raw, non-transformed signals, with a permutation order of 8 and a combination of AAFT and CPP surrogates (bolded F1 score). In other words, if a signal's permutation entropy (with a permutation order of 8) fell within the distributions of permutation entropies of *either* AAFT surrogate signals *or* CPP surrogate signals, generated from the raw original time-series, then that signal was very likely stochastic (either linear or nonlinear stochastic). Counter to prior expectations[1,15], no discernible benefit was gained by excluding signals classified as non-stationary by Breitung's Variance Ratio test. Table on next page.

Supplementary Table 2 (continued).

				n=3	n=4	n=5	n=6	n=7	n=8	n=9	n=10
Raw	Non-transformed	All data	AAFT	0.68	0.78	0.77	0.76	0.76	0.75	0.75	0.77
			FT	0.67	0.79	0.82	0.82	0.83	0.81	0.77	0.77
			CSS	0.01	0.01	0.02	0.04	0.08	0.07	0.19	0.29
			CPP	0.29	0.33	0.29	0.3	0.36	0.43	0.49	0.5
			IAAFT	0.67	0.73	0.77	0.77	0.77	0.74	0.71	0.7
			AAFT+CPP	0.68	0.86	0.86	0.86	0.87	**0.91**	0.89	0.89
		Stationary data	AAFT	0.59	0.72	0.71	0.7	0.7	0.69	0.69	0.72
			FT	0.58	0.72	0.76	0.76	0.77	0.75	0.71	0.7
			CSS	0.01	0.01	0.02	0.05	0.11	0.09	0.24	0.36
			CPP	0.28	0.32	0.28	0.28	0.36	0.46	0.53	0.55
			IAAFT	0.58	0.65	0.7	0.71	0.7	0.66	0.63	0.62
			AAFT+CPP	0.6	0.81	0.81	0.81	0.83	0.89	0.86	0.86
	Transformed (Box-Cox)	All data	AAFT	0.68	0.78	0.77	0.76	0.76	0.75	0.75	0.77
			FT	0.7	0.79	0.81	0.8	0.8	0.82	0.81	0.83
			CSS	0.03	0.03	0.03	0.04	0.07	0.04	0.1	0.12
			CPP	0.2	0.24	0.23	0.24	0.28	0.3	0.38	0.4
			IAAFT	0.65	0.7	0.72	0.73	0.74	0.75	0.76	0.75
			AAFT+CPP	0.59	0.74	0.76	0.76	0.77	0.78	0.77	0.78
		Stationary data	AAFT	0.59	0.72	0.71	0.7	0.7	0.69	0.69	0.72
			FT	0.61	0.72	0.74	0.74	0.74	0.77	0.76	0.77
			CSS	0.03	0.03	0.03	0.05	0.09	0.04	0.13	0.15
			CPP	0.16	0.21	0.2	0.21	0.25	0.27	0.37	0.38
			IAAFT	0.56	0.61	0.64	0.65	0.66	0.68	0.69	0.67
			AAFT+CPP	0.49	0.65	0.68	0.68	0.7	0.71	0.7	0.71
	Transformed (INT)	All data	AAFT	0.68	0.78	0.77	0.76	0.76	0.75	0.75	0.77
			FT	0.68	0.78	0.77	0.77	0.77	0.76	0.75	0.77
			CSS	0.06	0.1	0.15	0.27	0.13	0.02	0.1	0.09
			CPP	0.59	0.47	0.45	0.38	0.46	0.51	0.65	0.69
			IAAFT	0.67	0.8	0.8	0.79	0.8	0.79	0.79	0.8
			AAFT+CPP	0.67	0.73	0.76	0.75	0.77	0.78	0.78	0.78
		Stationary data	AAFT	0.59	0.72	0.71	0.7	0.7	0.69	0.69	0.72
			FT	0.59	0.72	0.71	0.7	0.7	0.69	0.69	0.72
			CSS	0.07	0.12	0.19	0.33	0.17	0.03	0.12	0.12
			CPP	0.51	0.39	0.39	0.27	0.33	0.37	0.54	0.57
			IAAFT	0.56	0.72	0.71	0.71	0.71	0.71	0.7	0.72
			AAFT+CPP	0.57	0.64	0.67	0.67	0.68	0.7	0.71	0.71
Denoised	Non-transformed	All data	AAFT	0.38	0.22	0.19	0.19	0.2	0.21	0.22	0.37
			FT	0.48	0.41	0.4	0.4	0.39	0.4	0.44	0.58
			CSS	0.05	0.15	0.12	0.12	0.14	0.17	0.11	0.3
			CPP	0.31	0.28	0.27	0.3	0.33	0.42	0.6	0.67
			IAAFT	0.47	0.47	0.47	0.47	0.48	0.5	0.54	0.59
			AAFT+CPP	0.51	0.44	0.42	0.44	0.47	0.55	0.7	0.78
		Stationary data	AAFT	0.43	0.25	0.23	0.24	0.24	0.26	0.26	0.43
			FT	0.48	0.42	0.42	0.42	0.42	0.43	0.46	0.59
			CSS	0.03	0.16	0.11	0.1	0.1	0.1	0.1	0.36
			CPP	0.32	0.31	0.32	0.35	0.38	0.48	0.61	0.72
			IAAFT	0.47	0.47	0.47	0.47	0.48	0.49	0.52	0.55
			AAFT+CPP	0.53	0.5	0.49	0.51	0.54	0.63	0.74	0.83
	Transformed (Box-Cox)	All data	AAFT	0.39	0.34	0.31	0.31	0.31	0.32	0.34	0.37
			FT	0.46	0.45	0.45	0.44	0.43	0.44	0.47	0.54
			CSS	0.02	0.13	0.1	0.13	0.17	0.1	0.16	0.18
			CPP	0.26	0.3	0.32	0.39	0.47	0.48	0.55	0.55
			IAAFT	0.5	0.49	0.51	0.52	0.52	0.54	0.56	0.6
			AAFT+CPP	0.45	0.5	0.49	0.55	0.61	0.63	0.67	0.66
		Stationary data	AAFT	0.44	0.41	0.38	0.38	0.39	0.4	0.41	0.43
			FT	0.49	0.5	0.51	0.49	0.49	0.5	0.53	0.57
			CSS	0.02	0.14	0.11	0.14	0.19	0.08	0.17	0.21
			CPP	0.19	0.24	0.27	0.35	0.44	0.42	0.48	0.5
			IAAFT	0.5	0.49	0.52	0.54	0.53	0.54	0.55	0.56
			AAFT+CPP	0.4	0.48	0.48	0.56	0.62	0.61	0.64	0.64
	Transformed (INT)	All data	AAFT	0.39	0.34	0.31	0.31	0.31	0.32	0.34	0.38
			FT	0.39	0.36	0.33	0.32	0.33	0.33	0.35	0.38
			CSS	0.04	0.08	0.13	0.1	0.03	0.04	0.05	0.09
			CPP	0.67	0.56	0.56	0.57	0.61	0.65	0.67	0.68
			IAAFT	0.43	0.43	0.42	0.41	0.42	0.44	0.47	0.51
			AAFT+CPP	0.71	0.65	0.66	0.7	0.75	0.78	0.78	0.78
		Stationary data	AAFT	0.43	0.4	0.38	0.38	0.38	0.4	0.41	0.43
			FT	0.44	0.42	0.4	0.39	0.4	0.41	0.42	0.43
			CSS	0.05	0.11	0.16	0.12	0.04	0.05	0.06	0.12
			CPP	0.55	0.47	0.46	0.45	0.47	0.5	0.54	0.55
			IAAFT	0.44	0.47	0.45	0.45	0.46	0.47	0.49	0.5
			AAFT+CPP	0.63	0.58	0.6	0.64	0.67	0.7	0.71	0.71

Supplementary Table 3: In Supplementary Table 2, we showed that the highest-performing surrogate-based method for detecting signal stochasticity was to use raw (non-denoised) signals, without normality transformation, and without excluding signals classified as non-stationary by Breitung's Variance Ratio test. For all analyses in Supplementary Table 2, we generated 500 surrogate time-series. Here, we tested whether generating 1,000 surrogate time-series from all raw time-series would yield higher performance in discriminating stochastic from deterministic processes. Though minor, we did observe higher F1 scores with a larger number of surrogates, and so the Chaos Decision Tree Algorithm uses 1,000 surrogates in its stochasticity test. We note that it is possible, and perhaps likely, that including even more surrogate signals in the stochasticity test will yield even more accurate results, and so the algorithm also allows the user to specify how many surrogates to generate.

	n=3	n=4	n=5	n=6	n=7	n=8	n=9	n=10
AAFT	0.67	0.79	0.78	0.77	0.76	0.76	0.75	0.78
FT	0.66	0.78	0.82	0.82	0.82	0.81	0.77	0.77
CSS	0.01	0.02	0.02	0.04	0.09	0.08	0.21	0.3
CPP	0.3	0.33	0.3	0.31	0.37	0.44	0.5	0.51
IAAFT	0.67	0.72	0.77	0.77	0.77	0.73	0.7	0.7
AAFT+CPP	0.67	0.86	0.87	0.87	0.88	**0.92**	0.89	0.89

Supplementary Table 4: To further test the relationship between stationarity and the accuracy of our surrogate-based stochasticity test, we broke down the performance of both Breitung's Variance Ratio test and our surrogate-based stochasticity test (which uses permutation entropy and a combination of Amplitude Adjusted Fourier Transform and Cyclic Phase Permutation surrogates) on all non-stationary processes analyzed in this paper, as well as stationary processes that are classically difficult to distinguish from non-stationary processes[3,4]. These included bounded random walks (which, though globally stationary, have local unit roots) and autoregressive processes with a moving average component. We found that our surrogate-based stochasticity test performed with near-perfect accuracy for *linear* stochastic non-stationary processes, i.e. random walks, trended random walks, and a cyclostationary autoregressive process, as well as for stationary linear stochastic processes with moving averages (which can be difficult to distinguish from non-stationary processes[3,4]). Where performance was slightly worse was for *nonlinear* stochastic processes, even though those processes were stationary (these processes were bounded random walks, the Freitas map, and the sine map). This suggests that non-stationarity may not affect the performance of surrogate-based stochaticity tests when such tests include non-Fourier based surrogates, such as the Cyclic Phase Permutation surrogates used here, though this possibility should be investigated more systematically in future work. In light of this result, and the results reported in Supplementary Table 2, the Chaos Decision Tree Algorithm does not automatically include a stationarity test, though the user can specify that the pipeline include a preliminary stationarity test and choose among any of the stationarity tests analyzed here.

	Fraction classified as stationary	Fraction classified as stochastic
Random walks (linear, stochastic, non-stationary)	23/500	498/500
Trended random walks (linear, stochastic, non-stationary)	16/500	477/500
Bounded random walks (nonlinear, stochastic, stationary)	487/500	451/500
ARMA(1) process (linear, stochastic, stationary)	488/500	481/500
Cyclostationary process (linear, stochastic, non-stationary)	500/500	498/500
Sine map (nonlinear, stochastic, stationary)	443/500	163/500
Freitas map (nonlinear, stochastic, stationary)	500/500	431/500

Supplementary Table 5: We tested the performance of three de-noising algorithms: a moving average filter (using Matlab's smooth.m function), Schreiber de-noising[16], and wavelet de-noising using an empirical Bayesian method with a Cauchy prior (using Matlab's wdenoise.m function). For each system, we created 100 noise-free simulations, and added white noise to those simulations, the amplitude of which was 40% the standard deviation of the original signals. We then applied the de-noising algorithms to the noise-contaminated signals, and calculated the Pearson correlation between the de-noised signals and the original noise-free signals. Shown: mean Pearson correlations across 100 datasets per system. Schreiber de-noising vastly outperformed the other two approaches, and so the Chaos Decision Tree Algorithm automatically uses Schreiber de-noising; the user can also specify that one of the other two de-noising methods be used.

System	Moving Average	Schreiber	Wavelet
Cortical model (chaotic)	0.81	0.96	0.90
Cortical model (periodic)	0.52	0.97	0.75
Spiking neuron (chaotic)	0.45	0.97	0.85
Granulocyte levels (chaotic)	0.76	0.94	0.87
Granulocyte levels (periodic)	-0.70	0.97	0
NF-κB transcription (chaotic)	0.97	0.99	0.99
NF-κB transcription (periodic)	0.97	0.98	0.97
Cubic map (chaotic)	0.090	0.95	0.020
Cubic map (periodic)	0.010	0.97	0
Cubic map (SNA HH)	-0.25	0.96	0
Cubic map (SNA S3)	0.65	0.97	0
GOPY map (SNA)	0.18	0.94	0
Logistic map (chaotic)	0.42	0.97	0.030
Logistic map (periodic)	0.67	0.98	0
Lorenz system (chaotic)	0.42	0.93	0.07
Generalized Hénon map (hyperchaotic)	0.060	0.94	0.010
Freitas map	0.55	0.94	0.51
Noise-driven sine map	0.97	0.98	0.97

Supplementary Table 6: De-noising and downsampling improves 0-1 test performance on oversampled continuous data. Shown: mean K statistic (which is the output of the 0-1 test), with $\sigma = 0.5$ (see Methods, Supplementary Figure 4), with standard error, across 100 samples of three oversampled simulations (the oversampling statistic η, whose mean across 100 samples is reported on the left, is the difference between the global maximum and global minimum of the signal divided by the mean absolute difference between consecutive time-points in the data[17]. If $\eta > 10$, then the downsampling approach simply downsamples the data until $\eta \leq 10$ or until there are fewer than 100 time-points left in the downsampled signal). The 0-1 test was performed on each dataset without downsampling, with downsampling, and with only local minima and maxima of the signal (following an alternative approach suggested by Eyébé Fouda and colleagues[18] to improve 0-1 test performance, which a user can select to use instead of downsampling). Note that for noise-contaminated oversampled data, only Schreiber de-noising followed by downsampling brings the K statistic within ranges expected for periodic or chaotic systems (highlighted).

Noise-free	Raw	Downsampled	Local Minima/Maxima
Chaotic Lorenz system ($\bar{\eta}$=334)	-0.0008 +/- 0	0.998 +/- 0	0.962 +/- 0.002
Periodic transcription ($\bar{\eta}$=21)	0.21 +/- 0.026	0.088 +/- 0.01	-0.026 +/- 0
Chaotic transcription ($\bar{\eta}$=199)	0.997 +/- 0	0.996 +/- 0.001	0.914 +/- 0.01

40% noise, no denoising	Raw	Downsampled	Local Minima/Maxima
Chaotic Lorenz system ($\bar{\eta}$=17)	0.998 +/- 0	0.998 +/- 0	0.998 +/- 0.001
Periodic transcription ($\bar{\eta}$=12)	0.997 +/- 0	0.982 +/- 0.003	0.998 +/- 0
Chaotic transcription ($\bar{\eta}$=26)	0.998 +/- 0	0.997 +/- 0.001	0.998 +/- 0

40% noise, Schreiber denoising	Raw	Downsampled	Local Minima/Maxima
Chaotic Lorenz system ($\bar{\eta}$=46)	0.998 +/- 0	0.994 +/- 0	0.997 +/- 0
Periodic transcription ($\bar{\eta}$=22)	0.991 +/- 0	0.364 +/- 0.01	0.998 +/- 0
Chaotic transcription ($\bar{\eta}$=94)	0.998 +/- 0	0.996 +/- 0.001	0.998 +/- 0

Supplementary Table 7: Sampling statistic in empirical (i.e. non-simulated) deterministic datasets. Because $\eta < 10$ in these datasets, none of them would be downsampled by the Chaos Decision Tree Algorithm.

System	
Neuron integrated circuit (chaotic)	$\bar{\eta}$=1.4
Neuron integrated circuit (SNA)	$\bar{\eta}$=1.9
Neuron integrated circuit (periodic)	$\bar{\eta}$=1.7
Laser[19] (chaotic)	η=7.9
Stellar flux[20] (SNA)	η=7

Supplementary Table 8: Classification accuracy of BenSaïda's implementation of the Shintani-Linton neural network-based chaos-detection algorithm[21] in raw (non-denoised), deterministic, simulated datasets. Systems were classified as periodic if their estimated largest Lyapunov exponent was zero or negative, and otherwise were classified as chaotic. Note that performance seems to go up at higher noise levels for periodic systems. This is likely because the algorithm is misclassifying these datasets as noise; pure noise has an infinite largest Lyapunov exponent, but neural network-based largest Lyapunov exponent estimators can often assign negative Lyapunov exponents to noise.

System	Measurement noise level (% of std. dev.)				
	0%	10%	20%	30%	40%
Cortical model (chaotic)	49/100	100/100	100/100	100/100	100/100
Cortical model (periodic)	0/100	0/100	0/100	0/100	0/100
Spiking neuron (chaotic)	100/100	100/100	100/100	100/100	100/100
Granulocyte levels (chaotic)	0/100	8/100	1/100	2/100	0/100
Granulocyte levels (periodic)	0/100	39/100	99/100	100/100	100/100
NF-κB transcription (chaotic)	0/100	0/100	0/100	0/100	0/100
NF-κB transcription (periodic)	100/100	100/100	100/100	100/100	100/100
Cubic map (chaotic)	86/100	77/100	30/100	0/100	0/100
Cubic map (periodic)	0/100	0/100	22/100	98/100	100/100
Cubic map (SNA HH)	0/100	0/100	0/100	44/100	88/100
Cubic map (SNA S3)	95/100	98/100	100/100	100/100	100/100
GOPY map (SNA)	0/100	0/100	0/100	76/100	100/100
Logistic map (chaotic)	100/100	99/100	100/100	95/100	23/100
Logistic map (periodic)	0/100	0/100	0/100	5/100	4/100
Lorenz system (chaotic)	0/100	0/100	0/100	0/100	0/100
Generalized Hénon map (hyperchaotic)	100/100	100/100	100/100	100/100	100/100

Supplementary Table 9: Classification accuracy of BenSaïda's implementation of the Shintani-Linton neural network-based algorithm[21] in deterministic, simulated datasets after Schreiber de-noising.

System	Measurement noise level (% of std. dev.)				
	0%	10%	20%	30%	40%
Cortical model (chaotic)	66/100	0/100	0/100	0/100	0/100
Cortical model (periodic)	0/100	0/100	0/100	0/100	0/100
Spiking neuron (chaotic)	100/100	100/100	100/100	100/100	100/100
Granulocyte levels (chaotic)	0/100	7/100	7/100	1/100	0/100
Granulocyte levels (periodic)	0/100	0/100	2/100	83/100	100/100
NF-κB transcription (chaotic)	0/100	0/100	0/100	0/100	0/100
NF-κB transcription (periodic)	100/100	100/100	100/100	100/100	100/100
Cubic map (chaotic)	80/100	66/100	47/100	15/100	4/100
Cubic map (periodic)	0/100	0/100	0/100	0/100	0/100
Cubic map (SNA HH)	0/100	0/100	0/100	0/100	2/100
Cubic map (SNA S3)	53/100	70/100	88/100	83/100	96/100
GOPY map (SNA)	0/100	0/100	0/100	2/100	100/100
Logistic map (chaotic)	27/100	14/100	93/100	100/100	100/100
Logistic map (periodic)	0/100	0/100	0/100	0/100	0/100
Lorenz system (chaotic)	0/100	0/100	0/100	0/100	0/100
Generalized Hénon map (hyperchaotic)	100/100	100/100	100/100	100/100	100/100

Supplementary Table 10: Classification accuracy of the NOLDS Python library implementation of the Rosenstein algorithm[22] in raw (non-denoised), deterministic, simulated datasets. Datasets were classified as chaotic if their estimated largest Lyapunov exponent was positive, and periodic if their estimated largest Lyapunov exponent was zero or negative.

System	Measurement noise level (% of std. dev.)				
	0%	10%	20%	30%	40%
Cortical model (chaotic)	100/100	100/100	100/100	100/100	100/100
Cortical model (periodic)	100/100	100/100	100/100	100/100	100/100
Spiking neuron (chaotic)	0/100	0/100	0/100	0/100	0/100
Granulocyte levels (chaotic)	4/100	7/100	10/100	6/100	9/100
Granulocyte levels (periodic)	100/100	90/100	90/100	90/100	90/100
NF-κB transcription (chaotic)	100/100	100/100	100/100	100/100	100/100
NF-κB transcription (periodic)	0/100	0/100	0/100	0/100	0/100
Cubic map (chaotic)	100/100	100/100	100/100	100/100	100/100
Cubic map (periodic)	6/100	78/100	72/100	99/100	100/100
Cubic map (SNA HH)	0/100	86/100	100/100	100/100	100/100
Cubic map (SNA S3)	0/100	0/100	0/100	0/100	0/100
GOPY map (SNA)	0/100	18/100	0/100	0/100	0/100
Logistic map (chaotic)	29/100	23/100	19/100	29/100	42/100
Logistic map (periodic)	100/100	90/100	92/100	97/100	90/100
Lorenz system (chaotic)	2/100	0/100	0/100	1/100	1/100
Generalized Hénon map (hyperchaotic)	0/100	0/100	0/100	0/100	0/100

Supplementary Table 11: Classification accuracy of the NOLDS Python library implementation of the Rosenstein algorithm[22] in deterministic, simulated datasets after Schreiber de-noising. Datasets were classified as chaotic if their estimated largest Lyapunov exponent was positive, and periodic if their estimated largest Lyapunov exponent was zero or negative.

System	Measurement noise level (% of std. dev.)				
	0%	10%	20%	30%	40%
Cortical model (chaotic)	80/100	100/100	100/100	100/100	100/100
Cortical model (periodic)	100/100	100/100	100/100	100/100	100/100
Spiking neuron (chaotic)	0/100	0/100	0/100	0/100	0/100
Granulocyte levels (chaotic)	87/100	85/100	84/100	56/100	22/100
Granulocyte levels (periodic)	100/100	85/100	90/100	87/100	85/100
NF-κB transcription (chaotic)	0/100	0/100	0/100	0/100	0/100
NF-κB transcription (periodic)	100/100	100/100	100/100	100/100	100/100
Cubic map (chaotic)	100/100	100/100	100/100	100/100	100/100
Cubic map (periodic)	38/100	3/100	12/100	70/100	66/100
Cubic map (SNA HH)	0/100	1/100	6/100	42/100	88/100
Cubic map (SNA S3)	0/100	0/100	0/100	0/100	0/100
GOPY map (SNA)	0/100	33/100	0/100	0/100	0/100
Logistic map (chaotic)	23/100	18/100	17/100	14/100	24/100
Logistic map (periodic)	100/100	84/100	86/100	86/100	89/100
Lorenz system (chaotic)	1/100	2/100	2/100	0/100	0/100
Generalized Hénon map (hyperchaotic)	0/100	0/100	0/100	0/100	0/100

Supplementary Table 12: Classification accuracy of the NOLDS Python library implementation of the Eckman algorithm[23] in raw (non-denoised), deterministic, simulated datasets. Datasets were classified as chaotic if their estimated largest Lyapunov exponent was positive, and periodic if their estimated largest Lyapunov exponent was zero or negative. With the exception of the noise-free simulation of periodic granulocyte levels, the algorithm yielded positive Lyapunov exponents for *all* datasets, hence the seemingly perfect classification accuracy for chaotic systems and the total inaccuracy for periodic systems.

	Measurement noise level (% of std. dev.)				
System	0%	10%	20%	30%	40%
Cortical model (chaotic)	100/100	100/100	100/100	100/100	100/100
Cortical model (periodic)	0/100	0/100	0/100	0/100	0/100
Spiking neuron (chaotic)	100/100	100/100	100/100	100/100	100/100
Granulocyte levels (chaotic)	100/100	100/100	100/100	100/100	100/100
Granulocyte levels (periodic)	100/100	0/100	0/100	0/100	0/100
NF-κB transcription (chaotic)	100/100	100/100	100/100	100/100	100/100
NF-κB transcription (periodic)	0/100	0/100	0/100	0/100	0/100
Cubic map (chaotic)	100/100	100/100	100/100	100/100	100/100
Cubic map (periodic)	0/100	0/100	0/100	0/100	0/100
Cubic map (SNA HH)	0/100	0/100	0/100	0/100	0/100
Cubic map (SNA S3)	0/100	0/100	0/100	0/100	0/100
GOPY map (SNA)	0/100	0/100	0/100	0/100	0/100
Logistic map (chaotic)	100/100	100/100	100/100	100/100	100/100
Logistic map (periodic)	0/100	0/100	0/100	0/100	0/100
Lorenz system (chaotic)	100/100	100/100	100/100	100/100	100/100
Generalized Hénon map (hyperchaotic)	100/100	100/100	100/100	100/100	100/100

Supplementary Table 13: Classification accuracy of the NOLDS Python library implementation of the Eckman algorithm[23] in deterministic, simulated datasets after Schreiber de-noising. Datasets were classified as chaotic if their estimated largest Lyapunov exponent was positive, and periodic if their estimated largest Lyapunov exponent was zero or negative.

	Measurement noise level (% of std. dev.)				
System	0%	10%	20%	30%	40%
Cortical model (chaotic)	100/100	100/100	100/100	100/100	100/100
Cortical model (periodic)	0/100	0/100	0/100	0/100	0/100
Spiking neuron (chaotic)	100/100	100/100	100/100	100/100	100/100
Granulocyte levels (chaotic)	100/100	100/100	100/100	100/100	100/100
Granulocyte levels (periodic)	0/100	0/100	0/100	0/100	0/100
NF-κB transcription (chaotic)	100/100	100/100	100/100	100/100	100/100
NF-κB transcription (periodic)	0/100	0/100	0/100	0/100	0/100
Cubic map (chaotic)	100/100	100/100	100/100	100/100	100/100
Cubic map (periodic)	0/100	0/100	0/100	0/100	0/100
Cubic map (SNA HH)	0/100	0/100	0/100	0/100	0/100
Cubic map (SNA S3)	0/100	0/100	0/100	0/100	0/100
GOPY map (SNA)	0/100	0/100	0/100	0/100	0/100
Logistic map (chaotic)	100/100	100/100	100/100	100/100	100/100
Logistic map (periodic)	0/100	0/100	0/100	0/100	0/100
Lorenz system (chaotic)	100/100	100/100	100/100	100/100	100/100
Generalized Hénon map (hyperchaotic)	100/100	100/100	100/100	100/100	100/100

Supplementary Table 14: Spearman correlations between largest Lyapunov exponents and permutation entropy calculated from raw data (i.e. data that were not de-noised and downsampled). While the correlations are still strong and significant for the discrete-time logistic and Hénon maps, performance is very poor for the continuous Lorenz system and mean-field cortical model. This is to be expected, because permutation entropy is equivalent to Kolmogorov-Sinai entropy (which is upper-bounded by a system's positive Lyapunov exponents) for *discrete* systems[24], and downsampling is effectively a discrete mapping of a continuous process. This is the same reason that de-noising and downsampling improves performance of the 0-1 test for continuous systems (Supplementary Table 6). Note that largest Lyapunov exponents in the cortical model are rough approximations (see Methods). *** $p<0.001$ after Bonferroni-correcting for multiple comparisons to the same set of ground-truth largest Lyapunov exponents.

	Measurement noise level (% of std. dev.)				
System	0%	10%	20%	30%	40%
Logistic map	0.97***	0.94***	0.94***	0.94***	0.94***
Hénon map	0.93***	0.92***	0.92***	0.88***	0.87***
Lorenz system	0.76***	-0.72***	-0.66***	-0.55***	-0.42***
Cortical model	0.15***	0.28***	0.01	0.01	0.04
Neuron integrated circuit	0.93***				

Supplementary Table 15: Accuracy of the (automated) Chaos Decision Tree Algorithm for different variables of the multi-dimensional systems analyzed in this paper. For the Lorenz system, the Rössler system, the Ikeda map, the Hénon map, the periodic cubic map, the strange non-chaotic cubic maps, and the period-doubled cubic maps, classification accuracy was high for any variable in the system, for relatively low levels of measurement noise. For higher levels of measurement noise, performance dropped for individual variables of the Lorenz, Rössler, GOPY map, and strange non-chaotic cubic map systems.

System	Measurement noise level (% of std. dev.)				
	0%	10%	20%	30%	40%
Lorenz system, **x** variable (chaotic)	100/100	100/100	98/100	77/100	34/100
Lorenz system, **y** variable (chaotic)	100/100	100/100	99/100	75/100	44/100
Lorenz system, **z** variable (chaotic)	100/100	100/100	99/100	75/100	44/100
Rössler system, **x** variable (chaotic)	37/100	57/100	88/100	100/100	100/100
Rössler system, **y** variable (chaotic)	97/100	98/100	99/100	100/100	100/100
Rössler system, **z** variable (chaotic)	100/100	51/100	92/100	33/100	16/100
GOPY system, **x** variable (SNA)	100/100	100/100	1000/100	83/100	21/100
Ikeda map, **x** variable (chaotic)	100/100	100/100	100/100	100/100	100/100
Ikeda map, **y** variable (chaotic)	100/100	100/100	100/100	100/100	100/100
Hénon map, **x** variable (periodic)	100/100	81/100	100/100	100/100	100/100
Hénon map, **y** variable (periodic)	100/100	100/100	100/100	100/100	100/100
Cubic map, **x** variable (chaotic)	90/100	93/100	89/100	88/100	99/100
Cubic map, **x** variable (periodic)	100/100	100/100	99/100	97/100	72/100
Cubic map, **x** variable (SNA HH)	100/100	100/100	100/100	100/100	41/100
Cubic map, **x** variable (SNA S3)	100/100	100/100	100/100	100/100	0/100
Cubic map, **x** variable (period-doubled)	100/100	100/100	100/100	100/100	32/100

Supplementary Table 16: Accuracy of the (automated) Chaos Decision Tree Algorithm for 1,000 time-points. Some datasets led to numerical errors during surrogate data generation; for these, we show classification accuracy out of the datasets that did not lead to errors.

System	Measurement noise level (% of std. dev.)				
	0%	10%	20%	30%	40%
Cortical model (chaotic)	100/100	100/100	68/100	24/100	51/100
Cortical model (periodic)	100/100	100/100	100/100	100/100	32/100
Spiking neuron (chaotic)	69/100	77/100	85/100	84/100	25/100
Granulocyte levels (chaotic)	100/100	100/100	100/100	100/100	100/100
Granulocyte levels (periodic)	100/100	100/100	100/99	99/100	94/100
NF-κB transcription (chaotic)	95/100	68/100	55/100	40/100	23/100
NF-κB transcription (periodic)	0/100	0/100	0/100	0/100	2/100
Cubic map (chaotic)	100/100	100/100	100/100	100/100	99/100
Cubic map (periodic)	100/100	100/100	100/100	97/100	52/100
Cubic map (SNA HH)	100/100	100/100	100/100	100/100	100/100
Cubic map (SNA S3)	100/100	100/100	100/100	100/100	55/100
GOPY map (SNA)	0/100	0/100	0/100	0/100	0/100
Logistic map (chaotic)	100/100	94/100	55/100	12/100	4/100
Logistic map (periodic)	100/100	100/100	100/100	100/100	98/99
Lorenz system (chaotic)	0/100	1/100	3/100	0/100	0/100
Generalized Hénon map (hyperchaotic)	100/100	100/100	99/100	34/100	1/100
Freitas map (nonlinear stochastic)	100/100	100/100	99/100	99/100	100/100
Noise-driven sine map (nonlinear stochastic)	9/83	65/85	79/90	98/100	100/100
Bounded random walk (nonlinear stochastic)	100/100	100/100	100/100	100/100	100/100
Cyclostationary process (linear stochastic)	93/100	97/100	98/100	99/100	100/100
ARMA(1) process (linear stochastic)	66/100	97/100	100/100	100/100	100/100
Trended random walk (linear stochastic)	98/100	97/100	100/100	100/100	100/100
Random walk (linear stochastic)	100/100	99/100	100/100	100/100	100/100
Rössler system (chaotic)	18/100	23/100	54/100	78/100	25/100
Ikeda map (chaotic)	100/100	100/100	78/100	11/100	24/100
Hénon map (periodic)	100/100	100/100	100/100	100/100	100/100
Cubic map (period-doubled)	99/100	95/100	60/100	12/100	0/100
Poincaré oscillator (periodic)	100/100	100/100	100/100	100/100	100/100
Poincaré oscillator (quasi-periodic)	100/100	100/100	100/100	100/99	73/100
Poincaré oscillator (chaotic)	100/100	100/100	100/100	96/100	67/100
Multivariate AR model (linear stochastic)	100/100	100/100	99/100	100/100	100/100
Violet noise (linear stochastic)	100/100				
Blue noise (linear stochastic)	100/100				
White noise (linear stochastic)	100/100				
Pink noise (linear stochastic)	100/100				
Red noise (linear stochastic)	99/100				

Supplementary Table 17: Accuracy of the (automated) Chaos Decision Tree Algorithm for 5,000 time-points.

	Measurement noise level (% of std. dev.)				
System	0%	10%	20%	30%	40%
Cortical model (chaotic)	100/100	100/100	100/100	62/100	91/100
Cortical model (periodic)	100/100	100/100	100/100	100/100	100/100
Spiking neuron (chaotic)	94/100	93/100	98/100	99/100	45/100
Granulocyte levels (chaotic)	100/100	100/100	100/100	100/100	100/100
Granulocyte levels (periodic)	100/100	100/100	100/100	100/100	100/100
NF-κB transcription (chaotic)	97/100	98/100	98/100	99/100	99/100
NF-κB transcription (periodic)	100/100	100/100	100/100	89/100	100/100
Cubic map (chaotic)	100/100	100/100	100/100	100/100	100/100
Cubic map (periodic)	100/100	100/100	100/100	100/100	100/100
Cubic map (SNA HH)	100/100	100/100	100/100	100/100	99/100
Cubic map (SNA S3)	100/100	100/100	100/100	100/100	0/100
GOPY map (SNA)	29/100	21/100	6/100	2/100	14/100
Logistic map (chaotic)	100/100	100/100	100/100	100/100	92/100
Logistic map (periodic)	100/100	100/100	100/100	100/100	100/100
Lorenz system (chaotic)	70/100	59/100	42/100	18/100	4/100
Generalized Hénon map (hyperchaotic)	100/100	100/100	100/100	100/100	26/100
Freitas map (nonlinear stochastic)	97/100	100/100	97/100	96/100	87/100
Noise-driven sine map (nonlinear stochastic)	38/100	22/100	56/100	50/100	96/100
Bounded random walk (nonlinear stochastic)	98/100	97/100	99/100	100/100	100/100
Cyclostationary process (linear stochastic)	97/100	98/100	100/100	100/100	100/100
ARMA(1) process (linear stochastic)	72/100	99/100	100/100	100/100	100/100
Trended random walk (linear stochastic)	98/100	89/100	98/100	100/100	100/100
Random walk (linear stochastic)	100/100	98/100	100/100	100/100	100/100
Rössler system (chaotic)	37/100	55/100	91/100	99/100	100/100
Ikeda map (chaotic)	100/100	100/100	100/100	93/100	23/100
Hénon map (periodic)	100/100	100/100	100/100	100/100	100/100
Cubic map (period-doubled)	100/100	100/100	100/100	100/100	100/100
Poincaré oscillator (periodic)	100/100	100/100	100/100	100/100	100/100
Poincaré oscillator (quasi-periodic)	100/100	100/100	100/100	100/100	100/100
Poincaré oscillator (chaotic)	100/100	100/100	100/100	100/100	100/100
Multivariate AR model (linear stochastic)	100/100	100/100	100/100	100/100	100/100
Violet noise (linear stochastic)	100/100				
Blue noise (linear stochastic)	100/100				
White noise (linear stochastic)	100/100				
Pink noise (linear stochastic)	100/100				
Red noise (linear stochastic)	98/100				

Supplementary Table 18: Fraction of datasets of noise-driven chaotic systems classified as stochastic, for different levels of intrinsic noise and different system observables. For both the stochastic Lorenz and stochastic Rössler systems, the parameter A controls the amplitude of intrinsic white noise injected into the **x** variable of the system (see Methods). The **x** (i.e. noise-driven) variable of the stochastic Lorenz system is classified as stochastic for any level of intrinsic noise, and classifications of stochasticity became more frequent for the **y**, **z**, and **x+y** variables with higher levels of intrinsic noise, particularly in the presence of additional measurement noise. For the stochastic Rössler system, increasing levels of intrinsic noise in the **x** variable led to more frequent classifications of stochasticity in all variables; interestingly, although noise was injected into the **x** variable, classifications of stochasticity became particularly frequent in the **y**, **z**, and **x+y** variables as the level of intrinsic noise was increased, especially (again) in the presence of additional measurement noise. Note that the level of noise injected into the Rössler system was lower than the level of noise injected into the Lorenz system, as we found that higher levels of dynamic noise led to numerical errors in the integration of the Rössler system.

	Measurement noise level (% of std. dev.)				
System	0%	10%	20%	30%	40%
Stochastic Lorenz, **x** variable (A=.5)	61/100	85/100	92/100	98/100	100/100
Stochastic Lorenz, **y** variable (A=.5)	8/100	48/100	65/100	94/100	100/100
Stochastic Lorenz, **z** variable (A=.5)	0/100	21/100	30/100	53/100	76/100
Stochastic Lorenz, **x+y** variable (A=.5)	45/100	60/100	73/100	97/100	99/100
Stochastic Lorenz, **x** variable (A=1.5)	98/100	82/100	93/100	99/100	100/100
Stochastic Lorenz, **y** variable (A=1.5)	1/100	0/100	4/100	62/100	99/100
Stochastic Lorenz, **z** variable (A=1.5)	0/100	81/100	84/100	95/100	95/100
Stochastic Lorenz, **x+y** variable (A=1.5)	35/100	9/100	29/100	86/100	99/100
Stochastic Lorenz, **x** variable (A=2.5)	97/100	99/100	99/100	100/100	100/100
Stochastic Lorenz, **y** variable (A=2.5)	0/100	0/100	0/100	34/100	90/100
Stochastic Lorenz, **z** variable (A=2.5)	0/100	91/100	66/100	87/100	99/100
Stochastic Lorenz, **x+y** variable (A=2.5)	40/100	28/100	46/100	92/100	99/100
Stochastic Rössler **x** variable (A=.05)	0/100	0/100	0/100	0/100	71/100
Stochastic Rössler **y** variable (A=.05)	0/100	0/100	78/100	0/100	0/100
Stochastic Rössler **z** variable (A=.05)	0/100	50/100	80/100	99/100	99/100
Stochastic Rössler **x+y** variable (A=.05)	0/100	0/100	0/100	72/100	0/100
Stochastic Rössler **x** variable (A=.15)	0/100	0/100	0/100	0/100	0/100
Stochastic Rössler **y** variable (A=.15)	0/100	0/100	0/100	0/100	0/100
Stochastic Rössler **z** variable (A=.15)	8/100	99/100	100/100	100/100	100/100
Stochastic Rössler **x+y** variable (A=.15)	0/100	0/100	0/100	0/100	0/100
Stochastic Rössler **x** variable (A=.25)	0/100	0/100	7/100	38/100	62/100
Stochastic Rössler **y** variable (A=.25)	87/100	96/100	98/100	97/100	99/100
Stochastic Rössler **z** variable (A=.25)	3/100	99/100	100/100	99/100	100/100
Stochastic Rössler **x+y** variable (A=.25)	40/100	47/100	65/100	84/100	92/100

Supplementary Table 19: The results for the autoregressive moving-average (ARMA) processes in Supplementary Tables 1-4 and in Table 2 are for random values of the moving average parameter θ. We sought to further test whether the moving average parameter θ had any systematic effect on our algorithm's performance. Here, we set the parameter ϕ to 0.99 as we did in Supplementary Tables 1-4 and in Table 2, and tested our algorithm on ARMA(1) processes with four different values of θ: -0.5, 0, 0.5, and 0.9. 10,000 time-points were generated for each simulation. Performance was high for all parameters.

	Measurement noise level (% of std. dev.)				
System	0%	10%	20%	30%	40%
ARMA(1), θ=-0.5 (linear stochastic)	88/100	96/100	100/100	100/100	100/100
ARMA(1), θ=0 (linear stochastic)	71/100	97/100	100/100	100/100	99/100
ARMA(1), θ=0.5 (linear stochastic)	74/100	99/100	98/100	100/100	100/100
ARMA(1), θ=0.9 (linear stochastic)	90/100	98/100	100/100	100/100	100/100

SUPPLEMENTARY NOTES

GLOSSARY

Deterministic. A process is deterministic if, given the exact same initial conditions, it will always evolve over time in the exact same way (i.e. there is no randomness built in to its evolution).

Measurement noise. All empirical recordings are contaminated by some level of measurement or "observational" noise, which is noise that is not intrinsic to a system. In other words, a system could be entirely deterministic, but because of measurement error, a signal recorded from that system could be noisy. To simulate such measurement error, we added random white noise of varying amplitudes to the datasets in Tables 1-5 and Supplementary Tables 1-4, 8-19.

Dynamic noise. Some systems have noise *built in* to the dynamics of the system. Such dynamic or "intrinsic" noise could be negligible, in that it is washed out by a system's deterministic components (and the system can therefore be modeled, in theory, by deterministic equations). Other systems, on the other hand, are significantly affected by intrinsic noise. Such systems are considered **stochastic**: no matter their initial conditions, they will always evolve over time differently, because their dynamics have some intrinsic randomness. In single-neuron dynamics, for example, there may be inherent stochasticity because of the probabilistic gating of voltage-dependent ion channels[25], though such stochastic events may be "washed out" by predominantly deterministic processes on larger scales. Several such systems were analyzed in this paper, including the noise-driven sine map, the Freitas map, bounded random walks, random walks, a cyclostationary autoregressive process, an autoregressive moving-average process, a random multivariate autoregressive process, colored noise, the stochastic Lorenz system, the stochastic Rössler system, the North Atlantic Oscillation index, and essential and Parkinson's tremors. In general, it is difficult to distinguish these stochastic (i.e. intrinsically noisy) processes from deterministic processes that are contaminated by measurement noise; it is also difficult to distinguish either case from deterministic chaotic processes (see below for definition).

Linear. The state of a linear process is directly proportional to its inputs or previous state (e.g. $y = ax$).

Nonlinear. The state of a nonlinear process is *not* directly proportional to its inputs or previous state (e.g. $y = ax^2$).

Largest Lyapunov exponent. The largest Lyapunov exponent of a system quantifies the largest rate of divergence of initially infinitesimally close trajectories through **phase space** (see below for definition).

Chaotic. A system is chaotic if it is bounded, deterministic, nonlinear, and has a positive largest Lyapunov exponent, meaning that initially similar phase space trajectories diverge exponentially fast.

Periodic. A system is periodic if it is deterministic, nonlinear, and has a negative largest Lyapunov exponent, meaning that initially similar phase space trajectories remain close.

Quasiperiodic. The dynamics of a quasiperiodic system exhibit regular cycles like those of a periodic system; but, unlike a purely periodic system that stably revisits the same system states, quasiperiodic systems return to states that are similar but not identical to previous states. Quasiperiodic systems are also not chaotic, because they are not sensitive to initial conditions (i.e. they have a negative largest Lyapunov exponent).

Period-doubled. In many dynamical systems, modulation of a system parameter can lead to an abrupt change in the system's dynamics, such that it oscillates at twice its original period. These systems are periodic (i.e. they have a negative largest Lyapunov exponent).

Strange non-chaotic. A strange non-chaotic system has a strange (i.e. fractal) phase space attractor like a chaotic system, but a negative or zero largest Lyapunov exponent. It is generally difficult to experimentally distinguish strange non-chaotic systems from chaotic systems[26].

Hyperchaotic. A hyperchaotic system is a deterministic, nonlinear system with more than one positive Lyapunov exponent. These systems are generally difficult to distinguish from noise[27].

Colored noise. Colored noise refers to stochastic processes with a non-uniform power spectrum (i.e. different levels of power at different frequencies). It is difficult to distinguish colored noise from chaos[27,28].

Degree of chaos. The magnitude of a system's largest Lyapunov exponent quantifies its degree of chaos. Higher largest Lyapunov exponents indicate higher degrees of chaos, because

they indicate faster rates of divergence in phase space.

Nonlinear stochastic. A nonlinear stochastic system is a nonlinear system with randomness built in to its evolution over time, making it difficult to distinguish from chaotic systems[27].

Stationarity. A stationary process is one whose joint probability distribution is time-invariant; in other words, for a stationary process, statistical properties like mean and variance do not fluctuate over time.

Attractor. An attractor is the orbit in phase space toward which a deterministic system tends to evolve. The attractors of chaotic systems are called "strange attractors" because they have a fractal structure.

Phase space. A space representing all possible states of a system. A single point in phase space corresponds to a single state of the system. For example, for one particle, a single point in phase space determines that particle's location and momentum. A dynamical system produces, in general, trajectories in its phase space, i.e., the system's state changes with time.

Schreiber de-noising algorithm. Almost two decades ago, Schreiber introduced a simple nonlinear noise-reduction algorithm[16], which replaces each point in a time-series with the average value of that point's "neighborhood" in phase space (see above for definition of phase space). The algorithm first uses delay coordinate embedding to create a map that is topologically equivalent to a system's ground-truth phase space attractor (a very common procedure in nonlinear time-series analysis[29]). Each point's neighborhood in phase space is defined by the number of steps k in that point's past and the number of steps l in that point's future that are used to construct embedding vectors, as well as the radius r of that point's neighborhood in phase space. The parameters k and l are set to 1, and the radius r is set to the standard deviation of the time-series.

Surrogate testing. A common approach for testing if a given time-series reflects a deterministic process is to create *surrogates* of that time-series, which share some key features with the original time series, such as its power spectrum and amplitude distribution, but are otherwise stochastic[1,12]. A "test statistic" is then calculated for both the original time-series and for the set of surrogate datasets, and if the value of the test statistic for the original time-series

lies outside the distribution of values for the surrogate datasets, then the original time-series likely reflects a deterministic process[1,12]. We follow Zunino and Kulp[30] and use permutation entropy as our test statistic (see below). We further tested a range of surrogate data generation algorithms, and picked a combination of Amplitude Adjusted Fourier Transform (AAFT) surrogates[12] and Cyclic Phase Permutation (CPP) surrogates[14], which led to the highest performance in detecting signal stochasticity (Supplementary Tables 2-3).

0-1 test for chaos. Gottwald and Melbourne's 0-1 test for chaos uses a given signal to drive a simple 2-dimensional system, and calculates the growth rate K of the mean square displacement of that system. K will approach 0 for periodic systems and will approach 1 for chaotic systems[26,31–34]. See Methods for more details. While the test has been used for some physics and engineering applications, it has seen only very limited application to biology[35].

Receiver operating characteristic (ROC) curve. An ROC curve assesses the accuracy of a binary classifier by plotting its true positive rate vs. false positive rate for different discrimination thresholds (in the case of the 0-1 test, the threshold in question is the cutoff for what K-statistic values that are classified as chaotic or as periodic). The more accurate a classifier is across discrimination thresholds, the larger its area under the curve in an ROC plot will be.

Permutation entropy. Permutation entropy is an extremely quick-to-compute and noise-robust measure of a signal's complexity[36]. Following Zunino and Kulp[30], the Chaos Decision Tree Algorithm uses permutation entropy to test if a time-series is deterministic or stochastic, by comparing the permutation entropy of a signal to the permutation entropies of its surrogates (Figure 1, Methods): if a given time-series reflects a predominantly deterministic process, then it will have a lower permutation entropy than its surrogates, since surrogates are inherently stochastic and therefore higher entropy than a matching deterministic (even deterministic chaotic) process. It's also for this reason that permutation entropy tracks degree of chaos[36,37], as stronger chaos means less predictability, and therefore more entropy. More formally, we should in general expect a close relationship between permutation entropy and systems' degree of chaos, because permutation entropy is equivalent to **Kolmogorov-Sinai entropy** for a broad class of discrete-time dynamical systems[24,38–40]. Kolmogorov-Sinai entropy is a mea-

sure of the entropy *rate* of a system, i.e. how much entropy a system generates per unit time. Kolmogorov-Sinai entropy tracks systems' degree of chaos, since it is upper-bounded by the sum of a system's positive Lyapunov exponents. This relationship is known as the "Pesin identity"[41]. While Kolmogorov-Sinai entropy is very difficult to measure from time-series data, its fast approximation through permutation entropy is not, making permutation entropy an ideal measure for the practical estimation of a system's degree of chaos. Although permutation entropy, like the 0-1 test, tracks chaos in discrete-time systems, we show that, just as with the 0-1 test, permutation entropy can also track chaos in *continuous* systems if signals from those systems have been properly downsampled (Table 5, Supplementary Table 14).

Supplementary References

1. Lancaster, G., Iatsenko, D., Pidde, A., Ticcinelli, V. & Stefanovska, A. Surrogate data for hypothesis testing of physical systems. *Physics Reports* (2018).
2. Timmer, J. Power of surrogate data testing with respect to nonstationarity. *Physical Review E* **58**, 5153 (1998).
3. Patterson, K. *Unit Root Tests in Time Series Volume 1: Key Concepts and Problems* (Springer, 2011).
4. Patterson, K. *Unit Root Tests in Time Series Volume 2: Extensions and Developments*, vol. 2 (Palgrave Macmillan, 2012).
5. Dickey, D. A. & Fuller, W. A. Distribution of the estimators for autoregressive time series with a unit root. *Journal of the American statistical association* **74**, 427–431 (1979).
6. Kwiatkowski, D., Phillips, P. C., Schmidt, P. & Shin, Y. Testing the null hypothesis of stationarity against the alternative of a unit root: How sure are we that economic time series have a unit root? *Journal of econometrics* **54**, 159–178 (1992).
7. Leybourne, S. J. & McCabe, B. P. A consistent test for a unit root. *Journal of Business & Economic Statistics* **12**, 157–166 (1994).
8. Lo, A. W. & MacKinlay, A. C. Stock market prices do not follow random walks: Evidence from a simple specification test. *The review of financial studies* **1**, 41–66 (1988).
9. Lo, A. W. & MacKinlay, A. C. The size and power of the variance ratio test in finite samples: A monte carlo investigation. *Journal of econometrics* **40**, 203–238 (1989).
10. Breitung, J. Nonparametric tests for unit roots and cointegration. *Journal of econometrics* **108**, 343–363 (2002).

11. Chan, K.-S. & Tong, H. *Chaos: a statistical perspective* (Springer Science & Business Media, 2013).

12. Theiler, J., Eubank, S., Longtin, A., Galdrikian, B. & Farmer, J. D. Testing for nonlinearity in time series: the method of surrogate data. *Physica D: Nonlinear Phenomena* **58**, 77–94 (1992).

13. Theiler, J. On the evidence for low-dimensional chaos in an epileptic electroencephalogram. *Physics Letters A* **196**, 335–341 (1994).

14. Jamšek, J., Paluš, M. & Stefanovska, A. Detecting couplings between interacting oscillators with time-varying basic frequencies: Instantaneous wavelet bispectrum and information theoretic approach. *Physical Review E* **81**, 036207 (2010).

15. Timmer, J., Häußler, S., Lauk, M. & Lücking, C.-H. Pathological tremors: Deterministic chaos or nonlinear stochastic oscillators? *Chaos: An Interdisciplinary Journal of Nonlinear Science* **10**, 278–288 (2000).

16. Schreiber, T. Extremely simple nonlinear noise-reduction method. *Physical Review E* **47**, 2401 (1993).

17. Matthews, P. 0 - 1 test for chaos. https://www.mathworks.com/matlabcentral/fileexchange/25050-0-1-test-for-chaos. (2009).

18. Eyébé Fouda, J. S. A., Bodo, B., Sabat, S. L. & Effa, J. Y. A modified 0-1 test for chaos detection in oversampled time series observations. *International Journal of Bifurcation and Chaos* **24**, 1450063 (2014).

19. Huebner, U., Abraham, N. & Weiss, C. Dimensions and entropies of chaotic intensity pulsations in a single-mode far-infrared nh 3 laser. *Physical Review A* **40**, 6354 (1989).

20. Lindner, J. F. *et al.* Strange nonchaotic stars. *Physical review letters* **114**, 054101 (2015).

21. BenSaïda, A. A practical test for noisy chaotic dynamics. *SoftwareX* **3**, 1–5 (2015).

22. Rosenstein, M. T., Collins, J. J. & De Luca, C. J. A practical method for calculating largest Lyapunov exponents from small data sets. *Physica D: Nonlinear Phenomena* **65**, 117–134 (1993).

23. Eckmann, J.-P., Kamphorst, S. O., Ruelle, D. & Ciliberto, S. Liapunov exponents from time series. *Physical Review A* **34**, 4971 (1986).

24. Amigó, J. M. The equality of Kolmogorov–Sinai entropy and metric permutation entropy generalized. *Physica D: Nonlinear Phenomena* **241**, 789–793 (2012).

25. White, J. A., Rubinstein, J. T. & Kay, A. R. Channel noise in neurons. *Trends in neurosciences* **23**, 131–137 (2000).

26. Dawes, J. & Freeland, M. The '0–1 test for chaos' and strange nonchaotic attractors. *preprint* (2008).

27. Kulp, C. & Zunino, L. Discriminating chaotic and stochastic dynamics through the permutation spectrum test. *Chaos: An Interdisciplinary Journal of Nonlinear Science* **24**, 033116 (2014).

28. Hu, J., Tung, W.-w., Gao, J. & Cao, Y. Reliability of the 0-1 test for chaos. *Physical Review E* **72**, 056207 (2005).

29. Sauer, T., Yorke, J. A. & Casdagli, M. Embedology. *Journal of statistical Physics* **65**, 579–616 (1991).

30. Zunino, L. & Kulp, C. W. Detecting nonlinearity in short and noisy time series using the permutation entropy. *Physics Letters A* **381**, 3627–3635 (2017).

31. Gottwald, G. A. & Melbourne, I. A new test for chaos in deterministic systems. In *Proceedings of the Royal Society of London A: Mathematical, Physical and Engineering Sciences*, vol. 460, 603–611 (The Royal Society, 2004).

32. Gottwald, G. A. & Melbourne, I. Testing for chaos in deterministic systems with noise. *Physica D: Nonlinear Phenomena* **212**, 100–110 (2005).

33. Gottwald, G. A. & Melbourne, I. On the implementation of the 0–1 test for chaos. *SIAM Journal on Applied Dynamical Systems* **8**, 129–145 (2009).

34. Gottwald, G. A. & Melbourne, I. Comment on "reliability of the 0-1 test for chaos". *Physical Review E* **77**, 028201 (2008).

35. Bernardini, D. & Litak, G. An overview of 0–1 test for chaos. *Journal of the Brazilian Society of Mechanical Sciences and Engineering* **38**, 1433–1450 (2016).

36. Bandt, C. & Pompe, B. Permutation entropy: a natural complexity measure for time series. *Physical review letters* **88**, 174102 (2002).

37. Trostel, M. L., Misplon, M. Z., Aragoneses, A. & Pattanayak, A. K. Characterizing complex dynamics in the classical and semi-classical duffing oscillator using ordinal patterns analysis. *Entropy* **20**, 40 (2018).

38. Politi, A. Quantifying the dynamical complexity of chaotic time series. *Physical review letters* **118**, 144101 (2017).

39. Bandt, C., Keller, G. & Pompe, B. Entropy of interval maps via permutations. *Nonlinearity* **15**, 1595 (2002).

40. Keller, K., Unakafov, A. M. & Unakafova, V. A. On the relation of KS entropy and permutation entropy. *Physica D: Nonlinear Phenomena* **241**, 1477–1481 (2012).

41. Pesin, Y. B. Characteristic Lyapunov exponents and smooth ergodic theory. *Russian Mathematical Surveys* **32**, 55–114 (1977).
